# Cement for Mending Rubber Goods

**Published:** 1898-09

**Authors:** 


### Cement for Mending Rubber Goods.

The following method of repairing the cracks or fissures in articles of rubber goods is given in *L'Industrie Textile.* First clean the surface of the fissure or parts to be united very carefully, and apply a cement composed of: Sulphide of carbon, 26 parts; gutta percha, 2 parts; caoutchouc, 4 parts; fish glue, 1 part. The edges of the rent should be kept together by means of thread and the article left to dry. At the end of from 24 to 36 hours the binding thread may be removed and the cement which may have squeezed out of the fissure cut away.

To MEND broken mortars first get the pieces thoroughly clean. Scrub with hot solution of soda and rinse well in warm water. Then, when dry, apply any of the following cements:

1. Melt together equal parts of shellac and gutta percha; heat the edges to be joined and apply the warm cement. This cement is elastic and resists the action of acids and weak alkalies.

2. Slake a good piece of quicklime with half its weight in water, and mix the dry hydrate with sufficient white of egg, previously beaten, to form a paste, and apply immediately.—*Merk's Report.*

